# Electrospun Ultrafine Cationic Cellulose Fibers Produced from Sugarcane Bagasse for Potential Textile Applications

**DOI:** 10.3390/polym13223927

**Published:** 2021-11-13

**Authors:** Andrés Felipe Ochica Larrota, Ricardo Vera-Graziano, Alex López-Córdoba, Edwin Yesid Gómez-Pachón

**Affiliations:** 1 Grupo de Investigación en Desarrollo y Nuevos Materiales-DANUM, Facultad de Ciencias Básicas Escuela de Ciencias Químicas, Universidad Pedagógica y Tecnológica de Colombia-UPTC, Tunja 150003, Colombia; andres.ochica@uptc.edu.co; 2 Instituto de Investigaciones en Materiales, Universidad Nacional Autónoma de México, Ciudad de México 04510, Mexico; graziano@unam.mx; 3 Grupo de Investigación en Bioeconomía y Sostenibilidad Agroalimentaria, Escuela de Administración de Empresas Agropecuarias, Facultad Seccional Duitama, Universidad Pedagógica y Tecnológica de Colombia, Carrera 18 con Calle 22, Duitama 150461, Colombia; alex.lopez01@uptc.edu.co; 4 Grupo de Investigación en Diseño, Innovación y Asistencia Técnica de Materiales Avanzados-DITMAV, Escuela de Diseño Industrial, Universidad Pedagógica y Tecnológica de Colombia-UPTC, Duitama 150461, Colombia

**Keywords:** electrospinning, fibers, cellulose, cationized cellulose, textile, dye

## Abstract

Sugarcane bagasse (SCB) is an abundant by-product of sugar refining that can be utilized as a raw material for cellulose isolation for several industrial applications. Electrospinning has garnered attention in recent years because it allows the preparation of cellulosic materials with unique properties. In this study, cellulose was isolated from sugarcane bagasse and acetylated to fabricate fine acetate cellulose fibers through electrospinning. Subsequently, the electrospun fibers were deacetylated and cationized in order to produce functionalized materials with potential textile applications. The functional fibers were colored with an anionic dye (vinyl sulfone) with and without the presence of salt and were evaluated according to dye fixation, color attributes, morphological characteristics, and thermal stability. Cationic cellulose fibers that were dyed without added salt were found to be brighter and demonstrated better color fixation than those with added salt. In addition, morphological analysis performed using scanning electron microscopy demonstrated that cationized fibers dyed without added salt were better preserved at this stage. The cationic fiber also evidenced a high-temperature resistance, exhibiting a degradation temperature above 236 °C. The results suggest that cellulose fibers dyed in this manner can potentially be considered for use in textile applications due to their suitable dye fixation and tunable porosity (i.e., breathability).

## 1. Introduction

Cellulose fibers are widely used in the textile industry as they are renewable, widely available, and have unique structural features, such as high mechanical strength, water holding capacity, low density, and dyeing ability, as well as their resistance to sunlight and repeated laundering. Cellulose fibers are also versatile materials that can be adapted by varied physical and chemical methods for use in different applications [1,2]. The main sources of cellulose are wood and cotton; however, a growing interest in the use of non-conventional cellulose sources, such as agricultural by-products, has been evidenced in the last few years [2,3].

Several methods for the production of cellulose textile fibers have been described [1,4]. Of these methods, electrospinning has become increasingly popular as it allows for cellulosic fibers to be prepared with unique properties [3,5]. For example, electrospun mats have been reported to afford increased breathability over conventional textiles due to the small pore size in between the fibers and their high porosity [5,6,7]. However, one of the key limitations affecting the production of cellulose textile fibers is their low solubility in common organic solvents and their inability to melt due to strong inter- and intra-molecular hydrogen bonds [3]. As a result, cellulose derivatives such as cellulose acetate have been extensively exploited due to their better solubility and electrospinnability [5].

In the textile industry, cellulose fibers are commonly dyed with reactive dyes. However, most of the dyes that are employed evidence a low color fixation with the cellulose fibers. This results in the need to utilize high concentrations of salt (30–100 g/L) during the dyeing process in order to improve the fiber–dye interaction [8]. This usage of salt results in significant environmental impacts due to the discharge of wastewater from the dyeing process that also has high levels of salt. It may be possible to mitigate these effects through the functionalization of cellulose by cationic groups, in combination with anionic dyes. This may improve dye fixation and has been evidenced to provide a wide range of bright colors, save costs, and increase color fastness in washing [6,7,9].

Sugarcane bagasse (SCB) is the expended cane fiber that remains after the process of extracting sugar juice has been completed. SCB is rich in cellulose (34–47%), hemicellulose (24–29%), and lignin (18–28%) [10]. Several methods have been reported to utilize this agro-industrial by-product as a raw material for cellulose isolation [10]. However, there are few studies that investigate the preparation and the dyeability of electrospun cationic cellulose fibers with textile applications, starting from sugarcane bagasse [7,11]. Approximately 270–280 kg of bagasse is produced from each ton of processed sugarcane [12]. Bagasse, as a waste product, is normally burned by the industry in order to produce the energy required in the extraction process.

The present study was targeted at the development of ultrafine electrospun cationized cellulose fibers from isolated cellulose by using chloride of 3-chloro-2-hydroxypropyl trimethylammonium (CHPTAC) as a cationizing agent. The fibers were colored in black with a vinyl sulfone anionic reagent. The structure and properties of the fibers were studied by Fourier-transform infrared spectroscopy (FTIR), scanning electron microscopy (SEM), differential scanning calorimetry (DSC), and thermogravimetric analysis (TGA). Moreover, color measurements and color fixation analysis were carried out to evaluate their potential use in textile applications. This study also intends to reduce the environmental impact of sugarcane bagasse waste.

## 2. Materials and Methods

### 2.1. Materials

Sodium hydroxide, NaOH (Emsure, 99 wt.%); nitric acid, HNO_3_ (Merck, 65 wt.%); and ethanol, EtOH (Sigma-Aldrich, 99.8 wt.%), were used to isolate cellulose. Disublimated iodine (Carlo Erba, 99.9 wt.%), acetic anhydride (Merck, 98.5 wt.%), and a microwave oven (SINEO MDS-8G) were used in the synthesis of cellulose acetate. *N*,*N*-dimethylformamide, DMF (Panreac, 99.8 wt.%), and acetone (Panreac, 93.5 wt.%) were used as solvents.

The cationization of cellulose fibers was carried out using a 3-chloro-2-hydroxypropyl trimethylammonium chloride solution (60 wt.% in H_2_O, Mw = 188) (Sigma Aldrich Inc., St. Louis, MO, USA). For dyeing fibers, a black coloring reagent (vinyl sulfone) (Sumicolor, Medellin, Colombia) was used. Sodium carbonate (99.99 wt.%), and anhydrous sodium sulfate (99 wt.%) were purchased from Sigma Aldrich (Sigma Aldrich Inc., St. Louis, MO, USA).

The sugarcane bagasse (SCB) was obtained from the agricultural industry present in the countryside of Moniquirá (Boyacá, Colombia). SCB was reported to have 46.7 ± 4.4 wt.% of cellulose; 19.7 ± 0.8 wt.% of lignin; 23.6 ± 2.1 wt.% of hemicellulose; and 8.8 ± 2.4 wt.% of other organic extractives [13].

### 2.2. Purification and Acetylation of Cellulose from Sugarcane Bagasse

The sugarcane bagasse (SCB) was washed three times with distilled water and dried at room temperature for 24 h. The raw material was first ground using a Samurai brand processor and then passed through an IKA A11B51 mill. Its subsequent purification was performed as described by Rodrigues Filho et al. [14,15]. Four grams of SCB was mixed with 76 mL of distilled water, which was then allowed to stand for 24 s. The mixture was then filtered and 76 mL of NaOH (0.25 M) was added. This was then allowed to rest for a further 18 h. The mixture was subsequently filtered again and the SCB was refluxed with three successive 100 mL portions of a solution of 20% HNO_3_/EtOH, where the solvent was changed hourly. After the reflux process, the mixture was filtered and washed with distilled water until the filtrate became colorless. The SCB was dried at 105 °C for 180 min and ground when dry, resulting in the production of purified sugarcane bagasse (PSCB).

The procedure reported by Li et al. [16] was used in the acetylation of PSCB, by mixing 0.8 g of cellulose, 20 mL of acetic anhydride, and iodine (15 mol%). The reaction was carried out at 130 °C for 30 min under 400 W microwave power (SINEO MDS-8G). Four replicates were prepared in the microwave tubes. The cellulose acetate from sugarcane bagasse (CASCB) that was produced was saved to produce fiber mats through electrospinning.

### 2.3. Electrospinning

The electrospinning equipment was assembled in the facilities of the Universidad Pedagógica y Tecnológica de Colombia. The equipment consisted of a variable voltage source (0–35 kV), a collector, and an injection pump (model NE- 4000, New Era Pump Systems, Inc., Farmingdale, NY, USA). The electrospinning process was conducted according to the protocol proposed by Khatri et al. [6].

A polymer solution of 17% by weight prepared in acetone/DMF (2:1) was pushed through a capillary tip and employed a syringe pump with a controlled feed rate. Electrical voltages in the range of 12 to 15 kV were applied to the capillary tip. The polymer ejected from the needle tip was collected on the collector. Table 1 shows the three sets of parameters used to prepare the CASCB fibers.

Deacetylation was carried out in a 0.05 M NaOH aqueous solution for 30 h at room temperature, after which it was thoroughly washed in distilled water until the fibers were at a neutral pH (pH = 7). Finally, all the cellulose fibers were dried at 50 °C for 4 h according to Khatri et al. [6,17]. The product of this reaction was called RCN (regenerated cellulose fibers).

The CASCB fibers, prepared according to the parameters described in Table 1, condition 3 (15 kV electrical voltage, 15 cm needle-collector distance, and 1000 μL/h injection rate), were chosen to be cationized following the protocol suggested by Wang et al. [8]. The fibers were continuously dipped in the aqueous solution of 3-chloro-2-hydroxypropyl trimethylammonium chloride (CHPTAC) at 8 wt.% and sodium hydroxide at 1.8 wt.% at room temperature, in a ratio of solution to fibers of 20:1. The obtained samples were heated at 60 °C for 6 min in an oven (MLW WSO 100 S1086). Finally, cationic cellulose fibers were thoroughly washed to remove traces of unfixed CHPTAC and were dried before the application of the reactive dye. The cationized fibers were called CCN (cationic cellulose fibers).

### 2.4. Fiber Dyeing

Condition 3 samples of fibers (Table 1) were dyed using a liquor to mats ratio of 50:1 The following procedures were carried out:

Procedure (a) for dyeing of non-cationized fibers (with added salt): The fibers were mixed with a 5% *w*/*v* dye solution and 2% *w*/*v* anhydrous sodium sulfate. After 10 min, sodium carbonate was added and heated for 60 min at 60 °C (MLW WSO 100 S1086 oven).

Procedure (b) for dyeing cationized fibers (without added salt): The fibers were mixed with a 5% *w*/*v* dye solution. After 10 min, sodium carbonate was added and heated for 60 min at 60 °C.

The product of this reaction was named DCN (dyed cellulose fibers).

### 2.5. Fiber Characterization

The degree of substitution (DS) was determined by a saponification reaction [15]. A blend of 5 mL NaOH (0.25 M) and 5 mL of ethanol was added to 0.1 g of cellulose acetate. The mixture was then left to rest for 24 h, before 10 mL of HCl (0.25 M) was added to the system and it was allowed to stand for a further 30 min. Then, the mixture was titrated in triplicate using a standard 0.25 M NaOH solution with phenolphthalein as an indicator. First, the percentage of acetyl group (% GA) was determined, as expressed by Equation (1) [15].

(1) $$GA=\frac{\left(\left(Vbi+Vbt\right)*µb-Va*µa\right)*43*100}{mCA}$$

Vbi = volume of NaOH added to the system; Vbt = volume of NaOH used in titration; µb = concentration of NaOH; Va = volume of HCl added to the system; µa = HCl concentration; 43 = molar weight of the acetyl group; mCA = weight of the cellulose acetate sample in mg.

Then, the DS was determined using Equation (2) [15].

(2) $$DS=\frac{3.86*GA}{102.4-GA}$$

DS = degree of substitution.

The reactions to prepare PSCB, CASCB, RCN, CCN, and DCN were monitored by attenuated total refraction Fourier-transform infrared spectroscopy (ATR–FTIR) using a NICOLET IS50 FT-IR Spectrometer. The morphology of the condition 1 and 2 fiber samples (Table 1) was studied by SEM (JEOL JSM-7600F), whilst the morphology of the condition 3 fiber samples was observed in the Zeiss-Evo MA10. The morphology, diameter, and porosity data were collected using Image-J software.

The thermal properties were determined by thermogravimetric analysis (TGA) and differential scanning calorimetry (DSC). The TGA analysis of the CASCB was performed with a TGA Q5000 V3.15 Build 263 TGA Module, whilst the TGA analysis of the DCN was conducted using a Q600 SDT V20.9 Build 20, DSC-TGA Standard Module, with both analyses being performed in a nitrogen atmosphere. The DSC analysis of the CASCB was conducted with a Build 122 V24.10 Q2000 DSC, DSC Module Standard Cell RC, while the DSC analysis of the DCN used a Q600 SDT V20.9 Build 20 with a DSC-TGA Standard Module. The thermal behavior of samples was recorded up to 400 °C at a heating rate of 10 °C/min under nitrogen atmospheric conditions.

Color measurements were conducted according to the NTMD-0151 Standard for both cellulose fiber samples dyed with salt and without salt. A Hunter Lab Ultra Scan VIS spectrophotometer under Illuminant D65 was used, employing a 10° standard observation with a UV component included, in wavelengths between 380 and 780 nm, and with the specular component excluded. CIELAB color coordinates (L*, a*, b*, C* and h*) were measured with QC Easy Match software by Hunter Lab. Color fastness tests were also carried out on cellulose fibers dyed with salt and cellulose fibers dyed without salt. The crocking test of the dyed cellulose fibers was carried out according to Colombian standard NTC-786, using a manual Crockmeter (SDL ATLAS, Rock Hill, SC, USA).

## 3. Results and Discussion

### 3.1. Acetyl Groups (%) and Degree of Substitution of Cellulose Acetate

The percentage of acetyl groups in the cellulose acetate was approximately 38.7% with a degree of substitution (DS) of around 2.3. The substitution yield was of the order of 70%. These results agree with the values previously reported in the literature [16].

According to Heinze et al. [18], the solubility of cellulose acetate in organic solvents is dependent on its degree of substitution. It has been reported that cellulose acetate with a degree of substitution ranging between 2.2 and 2.7 can be dissolved in acetone [18,19].

### 3.2. ATR–FTIR Studies

**Figure 1:**
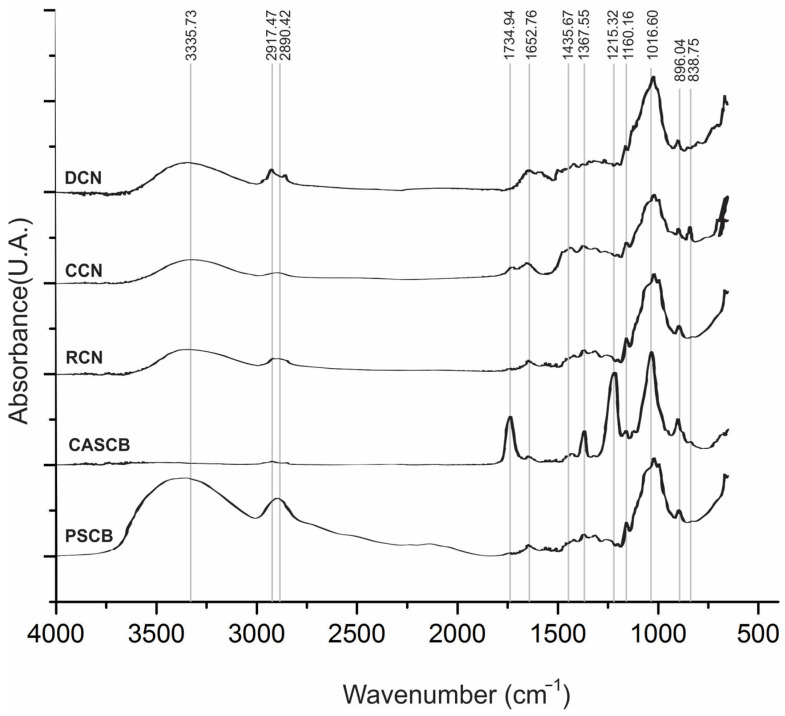
ATR–FTIR spectrum for cellulose samples obtained in the different stages of processing: pure sugarcane bagasse (PSCB), cellulose acetate from sugarcane bagasse (CASCB), regenerated cellulose fibers (RCN), cationic cellulose fibers (CCN), and dyed cellulose fibers (DCN).

Figure 1 shows the ATR–FTIR spectrum for pure sugarcane bagasse (PSCB), cellulose acetate from sugarcane bagasse (CASCB), regenerated cellulose fibers (RCN), cationic cellulose fibers (CCN), and dyed cellulose fibers (DCN). Results for PSCB show characteristic signals of cellulose at 3336 cm^−1^ (O–H stretching), 2917 cm^−1^ (C–H stretching), 1160 cm^−1^ (C–O stretching), and 1017 cm^−1^ (C–O stretching) [6].

The ATR–FTIR spectrum for CASCB showed characteristic vibrational bands of the acetate functional group of cellulose acetate at 1735 cm^−1^ (C = O stretching), 1367 cm^−1^ (C–CH_3_), and 1215 cm^−1^ (C–O–C). Similar results were reported by Rodrigues Filho et al. [15]. The RCN fibers showed the same vibrational bands of the cellulose (PSCB), indicating that the process used for the regeneration of cellulose was successful and that the electrospinning process did not generate further chemical changes. The results for CCN showed a signal at 1436 cm^−1^ that corresponded to the tension of the C–N group for cationizing (CHPTAC) and another band at approximately 839 cm^−1^ that can be attributed to the stretching of the corresponding epoxy ring [6]. DCN and CCN showed similar IR spectra, and new signals corresponding to the vinyl sulfone reactive dye were not detected. This behavior could be due to the low concentration of dye used in the dyeing process.

### 3.3. Fiber Morphology

**Figure 2:**
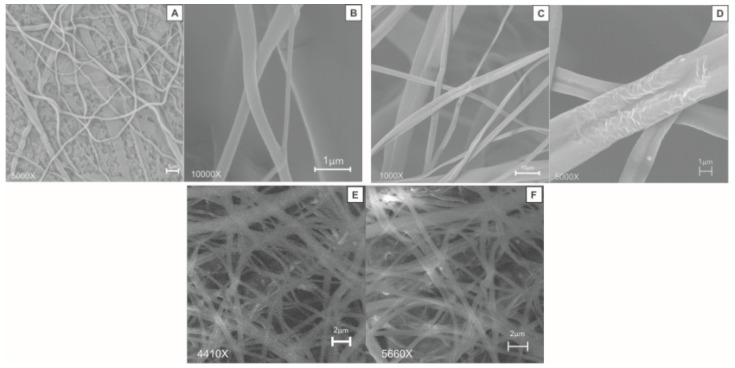
SEM micrographs of the fibers obtained using different electrospinning conditions (Table 1). Electrospinning conditions 1 (**A**,**B**), 2 (**C**,**D**), and 3 (**E**,**F**). Magnifications 5000× and 10,000×.

Figure 2 shows SEM micrographs of the fibers obtained using the different electrospinning conditions described in Table 1. In all three cases, the fibers formed porous mats of long fibers disposed randomly, without preferential orientation, and without uniform diameters.

Table 2 presents the results of the average diameter of the fibers and the porosity of the mats. The cellulose acetate fiber mats (CASCB) showed average diameters between 261 and 4452 nm and porosities between 20.8% and 35.5%. As can be observed, a needle-collector distance of 12 cm with electrical voltage of 12 kV and a flow rate of 3 μL/h (condition 1) presents the lowest fiber diameters (261 ± 123 nm) and the highest average porosity (35%). Under electrospinning conditions 2 and 3, the fibers showed higher average diameters and lower porosity. These results are important when considering that fiber diameter and web porosity have been reported to be closely related to the breathability of electrospun mats [20]. As such, higher breathability can be achieved by increasing the electrospun fiber diameter and pore size [20].

### 3.4. Thermal Stability

**Figure 3:**
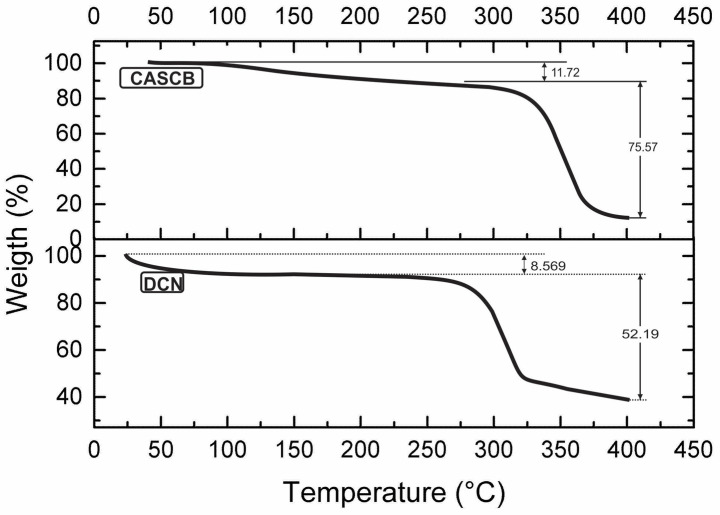
Thermograms of cellulose acetate from sugarcane bagasse (CASCB) and dyed cellulose fibers (DCN).

Weight loss as a function of temperature for CASCB and DCN is shown in Figure 3. A loss of 11.72% of weight in CASCB between 80 °C and 275 °C was attributed to the removed moisture, volatile substances, and initial degradation of lignin and hemicelluloses. Above 273 °C there is a continuous degradation of CASCB. In the case of DCN, a weight loss of 8.6% between 30 °C and 120 °C was attributed to removed moisture in the fibers. Above 236 °C there is a continuous degradation of the DCN.

**Figure 4:**
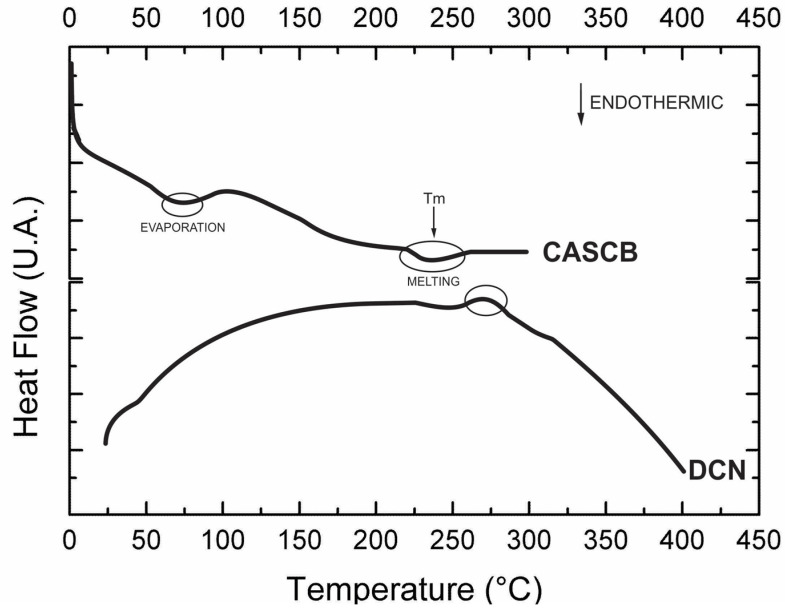
DSC of cellulose acetate from sugarcane bagasse (CASCB) and dyed cellulose fibers (DCN).

Figure 4 shows the DSC thermograms of cellulose acetate from sugarcane bagasse (CASCB) and dyed cellulose fibers (DCN). CASCB showed an endothermic peak at 74 °C that can be attributed to the evaporation of volatile substances and moisture [21]. In contrast, DCN does not show this enthalpic change due to the processes of cationization and dyeing, where, as the fibers are heated to 60 °C for 60 min, there is sufficient time and temperature conditions for the evaporation of volatile substances and water to occur.

The melting temperature of CASCB was observed to be approximately 235 °C with an enthalpic value of 4.9 J/g [21]. The enthalpic peak of DCN was recorded at 277 °C with an enthalpy value of 288.5 J/g, which may be due to polymer degradation, in accordance with the TGA results (Figure 3).

### 3.5. Color Attributes

The color attributes of the fibers colored with and without the presence of salt are shown in Table 3. Color differences were found between the samples, mainly in the values of b^*^ coordinate (i.e., blue-yellowness). The samples colored without the presence of salt shifted toward a bluer region in coordinate b*. In addition, these samples showed a lower hue angle (h) than the mats colored with the presence of salt (i.e., closest to the angle for blue (270°)).

**Figure 5:**
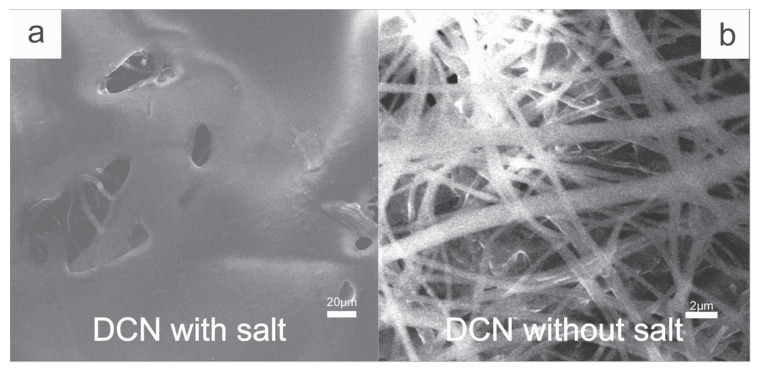
SEM images of dyed cellulose fibers (DCN) with salt (**a**) and without salt (**b**).

On the other hand, the results of the crocking test show that DCN without salt achieves a color transfer value of 5, whilst DCN with salt evidences a color transfer value of 4–5 (Figure S1, Table S1). A transfer value of 5 results in the best dye fixation and indicates that the process of cationization, dyeing without salt, makes the dye color fixation process more effective. Moreover, the results from the SEM micrographs (Figure 5) demonstrate that there was a greater color fixation during the process of cationization. As can be seen in Figure 5a, dyeing the fibers with salt does not result in an ideal fixation of the dye on the cellulose fibers, with tamponade of the fibers being evidenced. However, Figure 5b demonstrates that, in the case of cationized fibers, the fibers remain visible, which indicates that the dye has been fixed by the electrostatic interaction.

## 4. Conclusions

The present study presented the development of a process for the preparation of electrospun cationic cellulose fibers produced from sugarcanes bagasse, an industrial by-product, for use in textile applications. Cellulose was able to be successfully isolated from sugarcane bagasse and acetylated in order to fabricate electrospun acetate cellulose fibers. Electrospinning proved to be a simple and versatile method for producing fine acetate fibers (261–4452 nm) with tunable porosity (20.8–35.5%) and high thermal stability, which makes them suitable for textile applications.

Cationization proved to be an effective functionalization method to improve the dyeability of cellulose with anionic dyes such as vinyl sulfone. Cationic cellulose fibers that were dyed without added salt evidenced enhanced color fixation over fibers dyed with added salt. Despite the inclusion of a wet treatment during the dyeing process, the morphology of the fibers was also maintained at this stage of the process. These results demonstrate that the dyeing process of cationic cellulose fibers without added salt could potentially be used as a promising alternative to other methods. This may allow for a decrease in the salt levels of wastewater, making the dyeing process more sustainable and economically viable.

## Figures and Tables

**Table 1:** Electrospinning conditions used to obtain the cellulose acetate fibers.

Condition	Parameters		
	Tip-to-Collector Distance (cm)	Electrical Voltage (kV)	Feed Rate (μL/h)
1	12	12	3
2	14	15	8
3	15	15	1000

**Table 2:** Average diameter and porosity of the fibers obtained using different electrospinning conditions.

Condition	Average Diameter (nm)	Average Porosity (%)
1	261 ± 123 nm	35.5
2	4452 ± 2999 nm	30.0
3	678 ± 219 nm	20.8

**Table 3:** Color attributes of the fibers colored with and without the presence of salt.

CI Reactive	Fabric	L*	a*	b*	C*	h*
Black	DCN without salt	23.3 ± 0.4	3.8 ± 0.1	−1.5 ± 0.02	4.1 ± 0.2	338.5 ± 2.1
	DCN with salt	22.8 ± 0.5	4.2 ± 0.5	−0.22 ± 0.02	4.2 ± 0.2	356.9 ± 1.3

## Data Availability

The data presented in this study are available on request from the corresponding author.

## Supplementary Materials

The following are available online at https://www.mdpi.com/article/10.3390/polym13223927/s1, Figure S1: Color fixation test, Table S1: Color fixation according to NTC-786 standard.
