# Supplementary material for: Electrospun Ultrafine Cationic Cellulose Fibers Produced from Sugarcane Bagasse for Potential Textile Applications
Source: Polymers (Basel). 2021 Nov 13;13(22):3927. doi: 10.3390/polym13223927 (PMC8621662; doi:10.3390/polym13223927)
Supplement: Supplementary file 1 [file polymers-13-03927-s001.zip › polymers-1411474-supplementary.pdf]

polymers

Article

Electrospun Ultrafine Cationic Cellulose Fibers Produced from Sugar Cane Bagasse for Potential Textile Applications

Andrés Felipe Ochica Larrota [1], Ricardo Vera-Graziano[2], Alex López-Córdoba[3] and Edwin Yesid Gómez-Pachón[4]*

[1] Universidad Pedagógica y Tecnológica de Colombia-UPTC, Facultad de Ciencias básicas Escuela de Ciencias Químicas, Grupo de Investigación en desarrollo y nuevos materiales-DANUM. Tunja, Colombia. andres.ochica@uptc.edu.co
[2] Universidad Nacional Autónoma de México. Instituto de Investigaciones en Materiales, Ciudad de México, México. graziano@unam.mx
[3] Grupo de Investigación en Bioeconomía y Sostenibilidad Agroalimentaria, Escuela de Administración de Empresas Agropecuarias, Facultad Seccional Duitama, Universidad Pedagógica y Tecnológica de Colombia. Carrera 18 con Calle 22 Duitama 150461, Boyacá, Colombia; alex.lopez01@uptc.edu.co
[4] Universidad Pedagógica y Tecnológica de Colombia-UPTC, Escuela de Diseño Industrial. Duitama, Colombia, Grupo de Investigación en Diseño, Innovación y Asistencia Técnica de Materiales Avanzados-DITMAV. Duitama, Colombia edwin.gomez02@uptc.edu.co

* Correspondence: edwin.gomez02@uptc.edu.co; Tel.: 057-3107868975

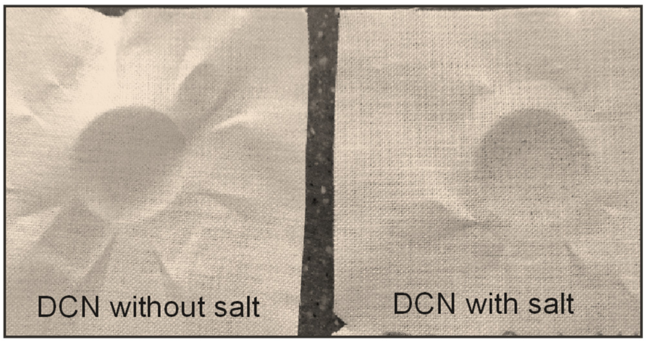

Figure S1. Color fixation test

Table S1. Color fixation according to NTC-786 standard

Dry Fixation	Color transfer
DCN without salt	5
DCN with salt	4-5
